# Aspects of Clinical Utility of the Distress Thermometer and Problem List after Burns

**DOI:** 10.3390/ebj3020027

**Published:** 2022-04-08

**Authors:** Helma W. C. Hofland, Anneke van de Steenoven, Nancy E. E. Van Loey

**Affiliations:** 1 Department Burn Centre, Association of Dutch Burns Centres, Maasstad Hospital, 3079 DZ Rotterdam, The Netherlands; loeyn2@maasstadziekenhuis.nl; 2 Department Burn Centre, Maasstad Hospital, 3079 DZ Rotterdam, The Netherlands; steenovenj@maasstadziekenhuis.nl; 3 Department Clinical Psychology, Utrecht University, 3584 CS Utrecht, The Netherlands

**Keywords:** screening instrument, burns, distress, effectiveness, relevance

## Abstract

Burn survivors may benefit from screening for a broad area of problems to improve communication and inform referral needs. Therefore, the aim of this study was to investigate clinical utility aspects such as appropriateness and acceptability to clinicians and completers of an existing, frequently used screening instrument in oncological populations, the Distress Thermometer and Problem List (DT and PL). Methods: Paediatric and adult patients visiting the outpatient clinic after admission to the burn centre were invited to complete the instrument. The DT and (problem domains of) the PL were related and compared to the need to discuss the reported problems. Results: A total of 160 patients were invited to complete the DT and PL, of which 139 agreed. The study shows evidence for appropriateness and high acceptability to clinicians and completers, although the effectiveness of the PL may be lower compared to the DT and needs adaptation to better meet the burn survivors’ situation. Discussion: The use of a screening instrument in the outpatient clinic environment has shown to be appropriate and acceptable and informs clinical practice to identify supportive needs in patients with burns. However, the PL needs to be adapted to the situation of the burn survivors.

## 1. Introduction

Burn survivors may be faced with a variety of physical and psychosocial problems following the burn event [1]. Among numerous physical problems, mental health problems are also common after burns but may receive less attention despite their large and long-term impact on health-related quality of life (HRQL) [2]. Several authors point to suboptimal psychological outcomes after burns and that more efforts are needed to improve psychological wellbeing [3]. For various reasons, e.g., time constraints, these problems may be left untouched by health care providers during the aftercare visits, and patients may not spontaneously raise all concerns [4,5]. A systematic screening of both physical and psychosocial functioning may help elucidate burn survivors’ concerns. Consequently, those can be discussed with the aim of lowering distress and identifying supportive care needs. If indicated, referral to other disciplines can be initiated with the purpose of improving physical and psychological outcomes.

The distress thermometer (DT) and problem list (PL) developed by the National Comprehensive Cancer Network (NCCN) [6] is a simple screening instrument that comprises a broad range of physical, social, psychological, practical, and spiritual problems. This instrument has been developed for patients with cancer with the aim of identifying personal needs and problem areas that may require help. The DT has been shown to be a valid and useful screening instrument [7,8,9]. The DT has also been validated in parents of children with a chronic disease [10]. Although the DT has been used beyond populations with cancer, the lack of validity studies currently prevents statements about its clinical utility beyond cancer populations [11]. The PL comprises items that screen for a range of problems and has shown to be a useful tool to elucidate problems that require attention. Advantages of the DT and PL are its use by nurses, and the instrument can also be used by caregivers [12]. The characteristics of the DT and PL, i.e., its brevity, ease of understanding, availability in multiple languages, and use for nurses and caregivers, increased the interest in testing the instrument for use in burn populations.

The use of broad screening instruments is not new and has recently also gained attention within the burns field. Gibson et al. [13] developed a burn-specific screening instrument, i.e., the Adult Burns Patient Concerns Inventory, that comprises four domains of functioning such as physical and functional well-being, psychological, emotional and spiritual well-being, social care, and social well-being, and treatment-related concerns. However, the instrument [13] does not comprise a distress measure rating scale which has shown to offer additional insight into the burden of the complaints.

The aim of this study was to explore the clinical utility of the Distress Thermometer and Problem List in the burn care population during outpatient clinic visits. Clinical utility comprises several components such as accessibility, practicality, appropriateness, and acceptability [11]. This study specifically piloted acceptability to clinicians and completers and appropriateness (effectiveness and relevance) of the DT and PL within the outpatient clinic environment.

## 2. Materials and Methods

This single-site study used a cross-sectional design. Adult burn patients, paediatric patients, and their caregivers visiting the outpatient clinic at the burn centre in Rotterdam, Netherlands, between December 2016 and April 2017 were asked to complete the DT and PL. The two inclusion criteria for participation were: acute burns as the primary diagnosis and admission to the Burn Centre. The exclusion criterion was insufficient Dutch proficiency.

The aftercare nurse orally informed the patients by introducing the instrument as a tool that measures a broad range of complaints that may occur in the aftermath of a burn injury. Subsequently, they were asked to complete the instrument. In the case of young children, caregivers were asked to fill in the DT and PL. Subsequently, they were asked if the patients or caregivers would care to talk about the problems and their distress. If they agreed, patients could have a conversation with the aftercare nurse. After their visit, the DT and PL was collected by the researchers for further analysis. The study was conducted according to the principles of the Declaration of Helsinki (revision, Fortaleza, Brazil, 2013). This study was approved by the Institutional Review Board of Maasstad Hospital, Rotterdam (Study number L2019092).

The distress thermometer and problem list (DT and PL) were used to measure potential distress and a variety of problem domains. The DT is a single tool that resembles a thermometer using a numeric rating scale ranging from 0 (no distress) to 10 (extreme distress). The DT and PL have been validated in Dutch [7]. The DT showed to be valid to measure distress in a population of patients with cancer [8]. A widely used cut-off point for clinically significant distress is ≥5. The DT has also been validated in parents of children with a chronic disease. For parents, a cut-off point of ≥4 has been proposed [14,15]. The PL consists of items allocated to five domains, including physical, emotional, family, social, practical, and spiritual problems [4]. For the purpose of this study, three items were added to the PL that were deemed relevant for (caregivers of) children with burns. These include feelings of guilt, loss of muscle strength, and condition. Feelings of guilt are commonly reported by parents [16,17], and also muscle strength and condition problems have been reported in children with burns [18]. The item regarding dry skin was adapted to itch, a well-documented problem for burn survivors [19]. Characteristics of the patient (i.e., gender and age) and the burn (i.e., percentage of total body surface area (TBSA) burned, number of surgeries during initial hospitalization) were reported from the medical file. TBSA burned is the estimated percentage of body surface area affected by partial and full-thickness burns.

All statistical analyses were conducted with IBM SPSS version 27. To compare respondents with non-respondents, chi-square tests and independent sample t-tests were performed. Pearson correlations coefficients were performed to investigate associations between the DT and problem domains. ANOVA was performed to compare the difference between three referral preferences (yes, maybe, and no) and the DT score and TBSA burned. The threshold for statistical significance was set at a two-tailed *p* < 0.05.

## 3. Results

### 3.1. Patient Characteristics

During the study period, 160 patients visiting the outpatient clinic were invited to complete the DT and PL, of which 139 participants filled in the DT and PL. of the 21 patients who did not fill in the instrument, 19 had low Dutch proficiency, and two refused. Of the 139 study participants, 48 were caregivers of children with burns, and 91 were adults. Children (0 to 16 years old) were on average 5.31 (SD = 4.02) years old, 22 (45.8%) were boys, TBSA burned was on average 6.03 (SD = 6.33) percent, 13 (27%) needed surgery. Adults were on average 43.55 (SD = 18.22) years old, 51 (56%) were male, Total body surface area (TBSA) burned was on average 6.75 (SD = 7.66), and the majority needed one or more surgeries (*n* = 51 (56%)).

### 3.2. Effectiveness of DT and PL Scores in Paediatric and Adult Burn Survivors

Evidence for appropriateness was evaluated by inspection of the DT score and the frequency of the items. The mean DT score was 2.75 (SD = 2.76) ranging from 0 to 8 in (caregivers of) children and 3.79 (SD = 2.72) ranging from 0 to 10 in adults. As presented in Table 1, the most frequently reported problems in both paediatric and adult respondents were emotional problems and physical problems. On average, the paediatric subgroup reported fewer problems (M = 4.96) compared to the adult subgroup (M = 9.34). In the paediatric subgroup, emotional problems that occurred in >15% included a lack of self-confidence, fears, a lack of concentration, and feelings of guilt. The most frequently reported physical problems were itchiness and difficulty sleeping. In the adult subgroup, problems occurred in the practical domain (housekeeping and work/school), emotional (i.e., feeling confused, forgetfulness, self-confidence, fears, depression, nervousness, concentration, loss of control), and the physical domain (i.e., appearance, pain, itch, sleep, weight changes, tingling, bathing, daily activities, fatigue, condition, muscle strength). Not all items were scored or only at a low frequency. This was particularly the case for several physical problems that have a low likelihood to occur after burns. Examples are changes in urination or problems swallowing.

In children, the DT correlated significantly with physical problems (r = 0.45) but not with other domains. In adults, the DT correlated with all domains, most strongly with the total score (r = 0.60), physical problems (r = 0.63), emotional problems (r = 0.55), practical problems (r = 0.55) (all *p* < 0.01), moderately with relational problems (r = 0.28), and social problems (r = 0.26) (*p* < 0.05). There was no statistically significant correlation with the DT and TBSA burned in both the paediatric and adult subgroup.

**Figure 1:**
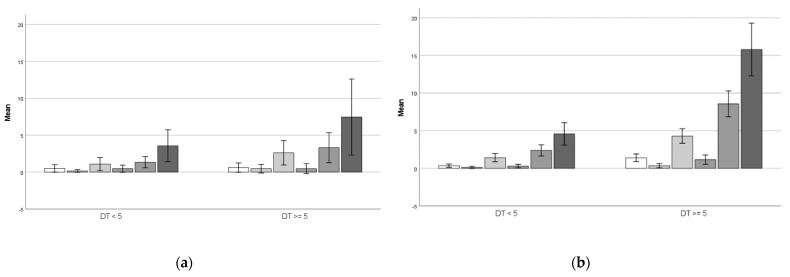
Bars represent the mean number of problems reported in the domains (from left to right): practical problems, social problems, emotional problems, religious concerns, physical problems, and total problems for respondents scoring above or below the cutoff point ≥5. (**a**) Pediatric respondents. (**b**) Adult respondents.

Using the cut-off point ≥ 5, 15 (25%) paediatric and 37 (30%) adult respondents, respectively, met this criterion. A cut-off ≥ 4 yields a proportion of 18 (37.5%) paediatric respondents who met this criterion of clinically relevant distress and 44 (48.4%) adult respondents. When using the DT cut-off ≥ 5 in relation to the total number of problems reported in every domain, Figure 1a (children) and Figure 1b (adults) show that respondents who score ≥5, report a higher number of problems compared to those scoring lower than five. However, in the paediatric subgroup, only physical problems were statistically significantly more frequently reported (t(46) = −2.59, *p* = 0.013), whereas in the adult subgroup, all domains excluding religious concerns, were statistically significant and more frequently reported (*p*-values < 0.01). This demonstrates that respondents who score ≥5 experience significantly more problems, particularly in the adult subgroup.

### 3.3. Relevance of DT and PL Scores in Paediatric and Adult Burn Survivors

**Figure 2:**
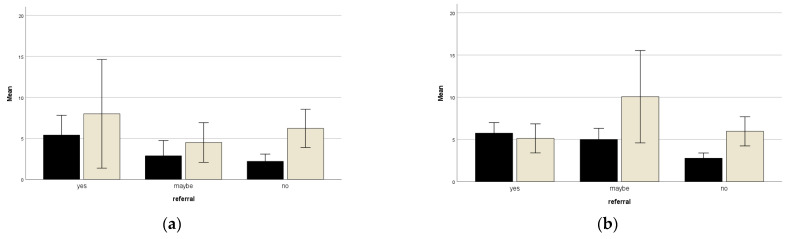
Bars representing mean DT scores and TBSA burned in relation to wish to discuss the problems (yes, maybe or not). Black bars represent mean DT scores, grey bars represent mean TBSA burned (**a**) Represents the result of the paediatric subgroup; (**b**) Represents the result of the adult subgroup.

Evidence for the relevance in clinical decision-making was assessed by investigating the number of study participants that wanted to discuss their problems with the aftercare nurse. Among the paediatric respondents, five (10.6%) wanted to discuss their problems with a health care provider, eight (17.1%) maybe wanted this, and 34 (72.3%) did not want to discuss it. In the adult group, 16 (17.8%) wished to discuss, 19 (21.1%) maybe wanted to discuss their problems, and 55 (61.1%) did not. Both paediatric and adult respondents who (maybe) wanted to talk about their problems had statistically higher scores on the DT compared to the respondents who choose not to discuss their problems (F_child_ (2, 44) = 3.34, *p* = 0.045; F_adult_ (2, 87) = 12.14 < 0.001). In contrast to the DT, the TBSA burned did not statistically differ across the three groups. Figure 2 shows the mean DT score and TBSA burned for the three groups.

### 3.4. Acceptability to Clinicians and Completers

As indicated by the high rate of respondents that were willing to complete the DT and PL, only two of 160 refused, and the acceptability showed to be high. The aftercare nurse also recognized the added value of a screening instrument that facilitated a focused approach and improved communication. However, not all items showed to have a relationship with the burn injury, indicating room for adaptation of the PL, for example, when respondents talked about constipation problems.

## 4. Discussion

In this study, aspects of the clinical utility, i.e., appropriateness (effectiveness and relevance) and acceptability, of a screening instrument developed for patients with a cancer diagnosis were tested in a group of paediatric and adult burn survivors. This study provided evidence for the effectiveness of the DT in both paediatric and adult respondents. Effectiveness may be lower for the PL, and the PL may be more tailored to the adult respondents’ health problems. Furthermore, this study provided evidence for relevance and showed to have good acceptability to completers and clinicians.

The DT showed to be appropriate to indicate the level of distress which was associated with the number of reported problems in the PL. The DT showed that adult burn survivors reported more distress and problems compared to the paediatric subgroup. When comparing the DT in adult burn survivors with adults with a cancer diagnosis, the burn population scored within the same range. In this study, 30% of the adult burn survivors scored ≥ 5 compared to 20.8 to 61.6% of patients within one year of a cancer diagnosis [20,21]. Distress is substantially higher in patient groups with cancer seeking support which was as high as 91% [12]. This indicates evidence of effectiveness.

Evidence of effectiveness may be lower for the content of the PL, and the instrument seems more useful in the adult sample compared to the paediatric sample. However, support for the PL as a measure to inform underlying problems of distress is also present in this study as the PL revealed that emotional and physical problems were most frequently reported and were most strongly related to the DT. This is in line with reports from adults with a cancer diagnosis [7]. However, the PL includes several items that did not apply to burn survivors, which calls for adaptation of the PL in the burns population. One should consider developing separate lists for paediatric (child and caregiver) and adult populations. Gibson et al. developed the Adult Burns Patient Concerns Inventory [13], which specifically addresses concerns and problems in the aftermath of burns. This screening instrument may inform a burn-specific problem list. Furthermore, also studies that include the patient perspective, such as Kool et al. study [1], can be used to determine relevant domains to screen for in burn survivors. The latter study indicates domains of functioning raised by burn survivors but needs to be transformed into a problem list that should be brief and easy to understand. To our knowledge, paediatric screening instruments, including both physical and psychological aspects, still need to be developed.

This study also provided evidence for relevance as it showed to inform clinical decision-making. More than one in three respondents (maybe) wanted to discuss their problems with a health care provider, which was linked to the level of distress and the number of problems they have to deal with in their lives. The results also showed that a cut-off point ≥ 5 may be useful in this adult population as both the ‘yes’ and ‘maybe’ subgroups had mean distress scores of five. In the paediatric respondents, a cut-off ≥ 5 may also apply. This would be higher than the earlier findings in parents of children with a chronic disease which suggests a cut-off ≥ 4 [15]. More research in burn populations is needed to establish cut-off points for clinically relevant distress. However, this study calls for the use of a screening instrument that includes a distress thermometer, adding value to estimate the burden of the complaints. Additionally, asking patients whether they wanted to discuss their problems was also shown to be an adequate indicator.

The study showed that the majority (139 (87%)) of burn survivors who visited the outpatient clinic and were asked to complete the instrument were willing to do, indicating good acceptability to completers. Language problems, which were described earlier as an important barrier to screening [22], were predominantly related to refusal to complete the instrument. Acceptability to clinicians was also high. The DT and PL informs the clinician whether the patient may benefit from psychosocial support and needs a referral. This subscribes to earlier reports [22] and shows that the DT and PL can assist nurse-guided follow-up, which may be particularly interesting for the burns field during outpatient clinic visits.

This study has some limitations that should be mentioned. First, it was not always clear whether the child or the caregiver had filled in the DT and PL. Therefore, the results of the paediatric subgroup should be interpreted with caution. Possibly, the psychological domain reflects the caregiver’s problems, which is suggested by the high rate of guilty feelings, while the physical problems likely reflect the child’s problems. A separate paediatric and caregiver version may be indicated when used in burn care practice. Second, the subgroups are relatively small, and therefore replication in a larger sample may be indicated.

## 5. Conclusions

In summary, this study provides the first indication that the use of screening instruments has clinical utility and that it is applicable to the context of burns. The majority of patients were willing to complete the tool, of which a substantial proportion had a number of problems they wanted to discuss with a professional. This subscribes to the notion that a screening instrument can help to identify the support needs of burn survivors, and it takes only a few minutes time. However, the DT with a cut-off point ≥5 should be further evaluated within the burns setting, but face validity is encouraging. The problem list needs adjustment to be tailored to burn-specific problems in both an adult and paediatric version.

## Figures and Tables

**Table 1:** DT and PL Frequencies, Mean (M) and Standard Deviations (SD).

	Child (*n* = 48)		Adult (*n* = 91)	
	N (%)	M (SD)	N (%)	M (SD)
DT		2.75 (2.76)		3.79 (2.72)
Total PL		4.69 (6.95)		9.34 (9.64)
Practical problems		0.56 (1.32		0.78 (1.23)
Child care	6 (12.5%)		6 (6.6%)	
Housing	2 (4.2%)		6 (6.6%)	
Housekeeping	4 (8.3%)		**17 (18.7%)**	
Transportation	2 (4.2%)		7 (7.7%)	
Work/school	5 (10.4%)		**20 (22.0%)**	
Finance	5 (10.4%)		9 (9.9%)	
Insurance	3 (6.3%)		6 (6.6%)	
Family problems		0.23 (0.67)		0.21 (0.64)
Dealing with partner	4 (8.5%)		3 (3.3%)	
Dealing with children	4 (8.5%)		7 (7.7%)	
Dealing with family/friends	3 (6.4%)		9 (9.9%)	
Emotional problems		1.51 (2.56)		2.53 (2.75)
Feeling confused	5 (10.6%)		**22 (24.2%)**	
Forgetfulness	7 (14.9%)		**39 (42.9%)**	
Self confidence	**8 (17.0%)**		**27 (29.7%)**	
Fears	**11 (23.4%)**		**23 (25.3%)**	
Depression	6 (12.8%)		**26 (28.6%)**	
Nervousness	7 (14.9%)		**33 (36.3%)**	
Feeling alone/isolate	5 (10.6%)		12 (13.2%)	
Concentration	**8 (17.0%)**		**21 (23.1%)**	
Feelings of guilt	**9 (19.1%)**		12 (13.2%)	
Loss of control	5 (10.6%)		**15 (16.5%)**	
Spiritual/religious concerns		0.46 (1.24)		0.63 (1.40)
Philosophy of life	3 (6.3%)		9 (9.9%)	
Relating to God	4 (8.3%)		8 (8.8%)	
Loosing sense of purpose	4 (8.3%)		9 (9.9%)	
Loss of faith	6 (12.5%)		10 (11.0%)	
Dealing with loss	3 (6.3%)		13 (14.3%)	
Facing mortality	2 (4.2%)		9 (9.9%)	
Physical problems		1.96 (2.75)		5.01 (4.96)
Appearance	7 (14.6%)		**32 (36.8%)**	
Changes in urination	0		7 (8.0%)	
Constipation	1 (2.1%)		4 (4.5%)	
Diarrhea	3 (6.3%)		9 (10.3%)	
Eating	7 (14.6%)		8 (9.2%)	
Feeling swollen	1 (2.1%)		9 (10.3%)	
Fevers	2 (4.2%)		6 (6.9%)	
Swallowing	0		4 (4.6%)	
Mouth sores	1 (2.1%)		4 (4.6%)	
Nausea	2 (4.2%)		10 (11.5%)	
Nose dry/congested	3 (6.3%)		11 (12.6%)	
Pain	5 (10.4%)		**35 (40.2%)**	
Sexual	0		8 (9.2%)	
Itch/dry skin	**23 (47.9%)**		**43 (49.4%)**	
Sleep	**11 (22.9%)**		**29 (33.3%)**	
Shortness of breath	0		12 (13.8%)	
Dizziness	1 (2.1%)		10 (11.5%)	
Talking	3 (6.3%)		8 (9.2%)	
Sense of taste	0		4 (4.6%)	
Weight changes	4 (8.3%)		**24 (27.6%)**	
Tingling in hands/feet	2 (4.2%)		**28 (32.2%)**	
Bathing/dressing	4 (8.3%)		**14 (16.1%)**	
Daily activities	3 (6.3%)		**14 (16.1%)**	
Fatigue	6 (12.5%)		**35 (40.2%)**	
Condition	4 (8.3%)		**37 (42.5%)**	
Muscle strength	1 (2.1%)		**31 (35.6%)**	
Other problems	1 (1.8%)		6 (6.9%)	

Note. Items scored in > 15% of the respondents were printed in bold.

## Data Availability

The raw data supporting the conclusions of this article will be made available by the authors upon request, without undue reservation.
